# Chia Seed Hydrogel as a Solid Fat Replacer and Structure Forming Agent in Gluten-Free Cookies

**DOI:** 10.3390/gels8120774

**Published:** 2022-11-28

**Authors:** Jelena Tomić, Dubravka Škrobot, Tamara Dapčević-Hadnađev, Nikola Maravić, Slađana Rakita, Miroslav Hadnađev

**Affiliations:** Institute of Food Technology, University of Novi Sad, 21000 Novi Sad, Serbia

**Keywords:** gluten free, chia seed, hydrogel, cookie, fat reduction

## Abstract

Gluten-free cookies based on rice and chickpea flour with reduced-fat and increased protein content compared with conventional commercial gluten-free cookies were developed and used as a base for further vegetable fat replacement with chia seed hydrogel. Rheological properties of chia seed hydrogel revealed that 8% gels exhibited the optimal properties as a fat substitute. Designed cookie samples were characterized for their chemical composition, fatty acid profile, mineral content, physical, textural and color parameters, and sensory properties. All gluten-free cookies developed in this study could be labeled as “a source of iron and potassium”, while those with chia seed hydrogel and cocoa powder could bear the additional claim “high in zinc and magnesium”. Fat replacement with chia seed hydrogel resulted in a more favorable fatty acid composition with a PUFA/SFA ratio over 0.40 and nonsignificant changes in the cookies’ hardness, weight, eccentricity, and specific volume, indicating that the chia seed hydrogel addition did not disturb the cookie structure and texture. The results of the sensory analysis confirmed that it is possible to apply chia seed hydrogel to produce reduced-fat cookies with sensory properties comparable to their full-fat counterpart and available commercial samples, and they are more appealing than commercial reduced-fat gluten-free cookies.

## 1. Introduction

Consumers’ interest and awareness concerning the health-enhancing role of specific foods or physiologically active food components pressurizes the food industry to continuously address dynamic consumer preferences and, consequently, reformulate commonly consumed food products. Special categories of food that require peculiar reformulation, which will result in a final product with an improved nutritional profile, are gluten-free products. A diet based on gluten-free products is highly recommended for individuals suffering from any form of gluten intolerance, especially celiac disease. Celiac disease is a chronic inflammatory intestinal disease (genetically determined) induced by gluten in wheat, barley, rye, etc. Scientific evidence regarding the nutritional profile of gluten-free products in comparison to their conventional counterparts confirmed that this category of products is often characterized by inferior nutritional quality. The main disadvantages of gluten-free products are thought to be the reduced dietary fiber and protein amounts, high fat and salt content, and increased glycemic index (GI) [1,2]. Therefore, commitment to a gluten-free diet can lead to a nutritional imbalance, which can increase the risk of diseases such as hypertension, diabetes, obesity, gastrointestinal and inflammatory disorder, food allergies, and intolerances [3]. Furthermore, gluten-free products are usually more expensive, with limited availability and poor sensory properties [4,5]. From the standpoint of sensory quality, gluten-free products based on conventional gluten-free flours and starches have inferior properties, especially in terms of aroma, texture, and taste. Therefore, it still remains a challenge for food processors to discover the optimal formulation with adequate ingredients capable of imitating the attributes typical for the gluten-containing counterparts [6,7]. Cookies, including gluten-free ones, are the most popular bakery snacks and an effective vehicle for supplying consumers with nutrients. Consequently, there are many studies dedicated to the nutritional improvement of gluten-free cookies through various research directions, such as fiber and protein enrichment or sugar and fat reduction [8,9,10]. Despite scientific and bakery community efforts to resolve problems regarding the quality of gluten-free products, there is still a need for further investigations in terms of enhancing nutritional and retaining or improving the technological quality of final products.

Chia seeds (*Salvia hispanica*) represent a novel food on the EU market whose quality requirements and application in different food categories, among which are baked and confectionary products, is regulated by the Commission implementing regulation [11]. Chia seeds have been recognized as a source of nutritionally valuable compounds, such as polyunsaturated fatty acids, vitamins, minerals, dietary fiber, proteins, and antioxidants, such as phenolic compounds [6,12,13]. Moreover, due to its high fiber content, chia seed gel possesses excellent technofunctional properties, such as hydration, viscosity development, and conservation of freshness, which could provide an opportunity to be used as a gluten mimetic as well as a fat replacer [14,15]. However, the production of cookies without gluten and with lower fat content encounters various technological problems, and the obtained products may have poor sensory properties [15,16].

Fat is an important component of cookies because it contributes to general appearance and texture and improves mouth feel and flavor [16,17]. There are a certain number of publications concerning the utilization of different types of gels, such as oleogels [18], hydrogels [19], bigels [20], and emulsion gels [21] to replace fat in energy-dense food products. The application of chia seeds in cereal-based products is most related to the evaluation of chia seed flour’s potential as a gluten-free ingredient in the production of various bakery products [22]. However, studies on the use of chia seeds gel as a fat replacer are quite rare and are mainly focused on gluten-containing cereal products [23,24]. 

Therefore, the aim of this study was to investigate the feasibility of chia seed hydrogels as fat replacer and gluten substitute in rice/chickpea flour-based gluten-free cookies with reduced fat content and an improved nutritional profile. The rheological properties of chia seed gels of different concentrations were investigated and used as a tool for the selection of appropriate gel concentration in further cookie formulation. Several cookie formulations with different vegetable fat/chia seed hydrogel were made, and their chemical and mineral composition, fatty acid profile, textural properties, eccentricity, specific volume, and sensory characteristics were studied. Commercial cookies were used as a benchmark in terms of the nutritional profile and sensory acceptability.

## 2. Results and Discussion

### 2.1. Chia Seed Hydrogel Rheological Properties

**Figure 1:**
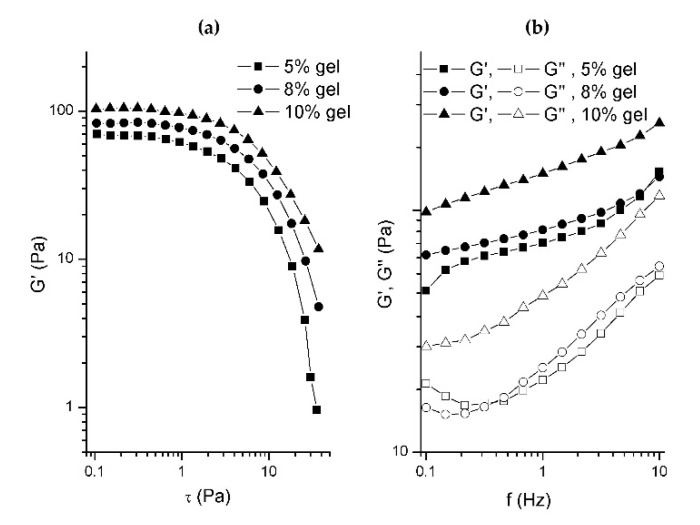
Rheological properties of chia seed hydrogels of different concentrations presented with (**a**) stress sweep and (**b**) frequency sweep curves.

In order to determine which concentration of chia seed hydrogel will be used in reduced fat cookie preparation, rheological properties of hydrogels were examined. Figure 1a represents stress sweep measurements, which were used to determine the viscoelastic region. The results of a dynamic oscillatory test (“frequency sweep” test), performed within the linear viscoelastic region, are shown in Figure 1b, and as it can be seen, all of the chia seed gel samples manifested viscoelastic behavior. All the samples exhibited a prevalence of an elastic (storage) modulus G’ over viscous (loss) modulus G’’, suggesting a viscoelastic solid behavior. As expected, the values of the elastic and viscous moduli were higher as the chia seed gel concentration increased, where the highest values were achieved in the sample with the highest chia seed concentration (10%). This observation is similar to the results of [25], where chia mucilage dispersions rheological properties were investigated. A common method to determine the viscoelastic behavior of the samples is by measuring the tangent of the phase angle (tan δ). A tan δ < 1 indicates a predominantly elastic behavior, whereas tan δ > 1 indicates a predominantly viscous behavior. The phase angle values (tan δ = G’’/G’), which were less than one, and a high frequency dependence of elastic and viscous moduli indicated that all the samples investigated in this study exhibited properties of weak gels. Comparing all three chia seed gel concentrations, 8% hydrogel had a characteristic behavior that was more similar to 10% hydrogel in terms of yield stress, i.e., the value of the stress at which deviation from the linear viscoelastic region occurs (Figure 1a), while in terms of the gel network structure organization and strength (Figure 1b), it resembled 5% hydrogel. On the contrary, 5% hydrogels were characterized with higher susceptibility to the permanent deformation of the structure (Figure 1a), while 10% hydrogels were too rigid to mimic the fat structure. This was confirmed with preliminary baking trials (results not shown) with all three hydrogels in which cookies with 8% chia seed hydrogels performed the best in terms of cookie quality attributes. Therefore, the 8% chia seed hydrogel was chosen as optimal for further experiments.

### 2.2. Proximate Composition of Gluten-Free Cookies

The chemical composition of the gluten-free cookies developed in this study is presented in Table 1 along with the information about the chemical composition of commercially available gluten-free cookies (Com 1—based on a mixture of buckwheat, maize, millet, and rice flours; Com 2—based on rice flour with the addition of plant proteins; Com 3—based on maize and rice flours with the addition of almond; and Com 4—based on millet flour with the addition of cocoa powder) obtained from their nutrition labelling declarations. The chosen commercial gluten-free cookies represent diverse types of cookies in terms of the basic ingredients used for formulation, their nutritional profile, and popularity among consumers. The proximate composition of commercial gluten-free cookies was used as a starting point to create a novel cookie formulation with lower fat content. After determining the concentration of the chia seed gel, which is used as a fat replacer, the ratio of chia seed gel and vegetable fat in the formulation varied. Additionally, in the CHC1 and CHC2 samples, cocoa powder was added. The above-mentioned formulation modifications affected the chemical composition of the examined cookies. The cookies developed in this study, including the Control one, had a higher content of proteins than Com 1, Com 3, and Com 4 due to the chickpea flour addition, as well as a lower content of fat compared to the Com 2–4 commercial samples, which mostly resulted in cookies with a reduced energy value. On the other hand, the cookies developed in this study had higher sugar and lower fiber content compared with commercial samples, since in commercial samples a high fiber content was influenced with the presence of wholegrain flour. In general, according to Regulation (EC) No 1924/2006 [26], the Control and CH, CHC1, and CHC2 cookies could bear a nutrition claim of “reduced fat” since the reduction in fat content was at least 30% compared with the commercial products Com 2–4. In the cookies with chia seed hydrogel, the highest fat reduction (18.4%) compared with the Control one was achieved in the CHC2 sample. In addition to the expected fat reduction, the presence of chia seed gels influenced the increase in fiber and ash content and the reduction in saturated fatty acids, while the carbohydrate content remained almost unchanged compared with the Control sample. These results suggest that further pathways in formulation improvement could be focused on the reduction in raw materials that contain a large share of carbohydrate components, such as a commercial gluten-free mixture.

### 2.3. Fatty Acid Composition of Gluten-Free Cookies

Fatty acids play a significant role in the prevention of many chronic diseases. A balanced diet rich in omega-3 fatty acids results in health benefits related to cardiovascular disease, inflammation, hyperlipidemia, and cancer prevention [27]. Plant sources such as chia, perilla, buckwheat, and flax seeds are recognized as useful sources of fatty acids [28,29]. Table 2 presents the fatty acid profile of prepared gluten-free cookies. In all cookie samples, palmitic (C16:0), *cis*-oleic acid (C18:1c), and *cis*-linoleic acid (C18:2c LA) were the most abundant fatty acids. A similar trend was obtained in another study [30], where oat-based gluten-free cookies with different types of fat were examined. According to WHO/FAO, a balanced diet should be characterized by a PUFA/SFA ratio higher than 0.4 [31]. All the reduced-fat gluten-free cookie samples in this study had a PUFA/SFA ratio over 0.4, which means that the vegetable fat substitution with chia seed hydrogel contributed to a favorable fatty acid composition. Moreover, vegetable fat reduction with a higher amount of chia seed hydrogel (CHC2) led to a decrease in the content of saturated fatty acids, while the amount of polyunsaturated fatty acids remained the same.

### 2.4. Mineral Content of Gluten-Free Cookies

The mineral content of the gluten-free cookies is shown in Table 3. The substitution of vegetable fat with chia seeds gel significantly (*p* < 0.05) altered the content of minerals. When compared with the Control, the gluten-free cookies with chia seed hydrogel had an increased content of minerals, whereas the contribution of cocoa powder was reflected in the increase in Mg, Fe, and Zn content. The obtained results are significant bearing in mind that gluten-free products have a low amount of minerals, which can be harmful for patients suffering from celiac disease [4,15]. To label a product as the one with a significant amount of certain minerals, it must contain at least 15% of its nutritional referent value (NRV) in 100 g of product. It can be concluded that all gluten-free cookie samples developed in this study could be labeled as “a source of iron and potassium”, while CHC1 and CHC2 can be additionally labeled as “high in zinc and magnesium”.

### 2.5. Textural Properties of Gluten-Free Cookies

**Figure 2:**
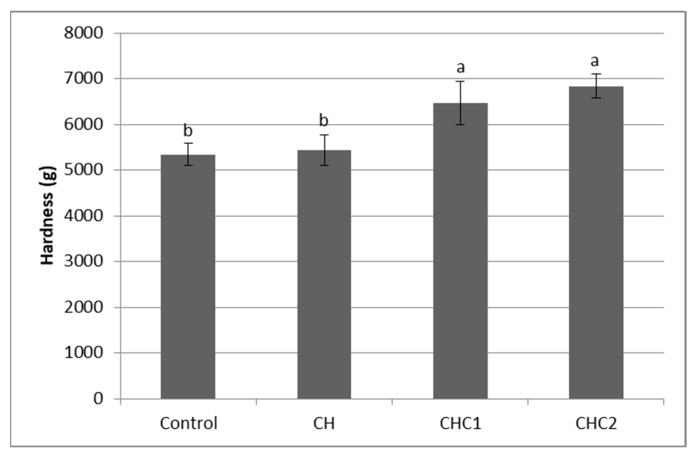
Hardness of developed reduced-fat gluten-free cookies (CH, CH1, CH2) compared with full-fat cookie (Control). Different small letters indicate statistical significance (*p* < 0.05).

Fat is one of the major ingredients that affect cookie texture. The substitution of fat has a greater impact on the textural attributes of cookies than sugar or flour replacement [33]. The formulation of cookies, which can simulate their high-fat counterparts, must be carried out carefully in order to obtain a final product with acceptable textural properties [16,17]. Figure 2 illustrates the results of cookies hardness. Hardness refers to how easily the product can be break by compression [34]. Fat reduction in cookies formulation by chia seed hydrogel resulted in a slight increase in cookie hardness, which was not significant between the samples with the same amount of cocoa powder. However, the addition of cocoa powder, which was made in order to improve the cookies’ sensory properties, significantly (*p* < 0.05) contributed to the hardness increase (CHC1 and CHC2 cookies). Mostly, water-absorbing components such as fiber and protein contribute to the hardness increase [34]. Contrary to these, [35] found that a xanthan gum gel addition in the formulation of gluten-free cookies resulted in higher dough hardness but reduced cookies hardness. It was explained by the exudate gum’s ability to retain moisture within the cookies, which contributed to a decrease in hardness. In this study, chia seed hydrogel was able to retain water and provide an acceptable structure to gluten-free cookies, while the addition of cocoa powder led to changes in water distribution, which contributed to the hardness increase.

### 2.6. Physical Properties and Color of Gluten-Free Cookies

**Figure 3:**
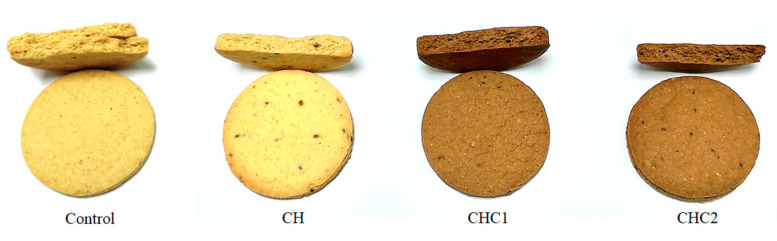
Appearance of developed reduced-fat gluten-free cookies (CH, CH1, CH2) and full-fat cookie (Control).

The results presented in Table 4 indicate that there were no significant differences between the cookie samples in terms of mass, eccentricity, and specific volume, indicating that both the chia seed hydrogel and cocoa powder addition did not disturb the cookie structure. This could be confirmed with the cookie appearance presented in Figure 3, which revealed that the reduced-fat cookie samples did not differ from the Control in terms of spread, surface crack, cross section structure, and porosity. 

The differences between the color of the cookies, Control, and CH vs. CHC1 and CHC2 (Figure 3, Table 4) are the consequence of the difference in the composition of the gluten-free cookies, i.e., the cocoa powder addition. Among the samples with the same cocoa powder content, a statistically significant difference (*p* < 0.05) in parameter b* was noticed between the CHC1 and CHC2 samples (Table 4). The obtained findings can be explained with the different fat composition in the cookie preparations, where the samples with a lower fat content had a higher share of a yellow color.

### 2.7. Sensory Acceptability of Gluten-Free Cookies

**Figure 4:**
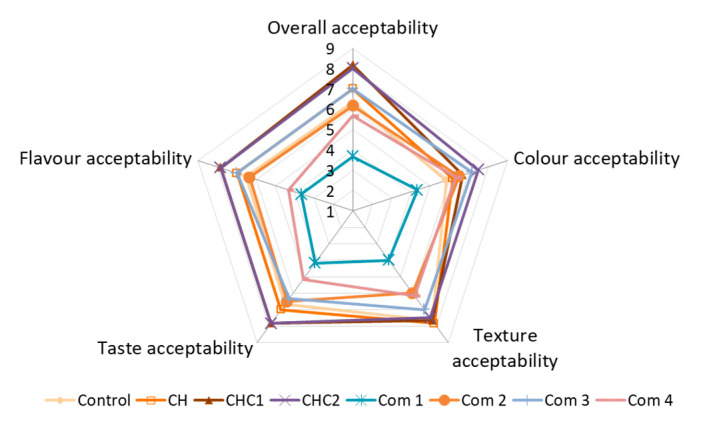
Overall acceptability of developed reduced-fat gluten-free cookies (CH, CH1, CH2), compared with full-fat cookie (Control).

The impact of the chia seed hydrogel fat replacer on the overall acceptability and acceptability of the color, taste, flavor, and texture of gluten-free cookies, evaluated by consumers, is shown in Figure 4. The cookies formulated in this study were compared with each other as well as with commercially available gluten-free cookies. Scores for overall acceptability were in the range from 3.67 to 8.17, which means that consumers discriminated cookies from those that are disliked slightly (Com 1) to those that are liked very much (CHC1). The decrease in fat content in the produced samples did not impair the acceptability of the cookies’ texture nor flavor. A similar finding was reported by [23] who investigated the influence of a partial substitution of fat with chia gel mucilage and concluded that cookies with up to 30% fat substitution were found acceptable. However, according to these authors, higher substitution levels caused a reduction in the sensory acceptability of the cookies. The addition of cocoa powder in the gluten-free cookies’ formulation (CHC1 and CHC2 cookies) contributed to a higher acceptability of all the analyzed sensory properties, except the texture. In general, the results of the sensory analysis revealed that it is possible to apply chia seed hydrogel to produce reduced-fat cookies with sensory properties comparable to their full-fat counterparts (Control) and commercial samples (Com 2 and Com 3), and that they are far more appealing than commercial low-fat gluten-free cookies (Com 1).

## 3. Conclusions

The utilization of chickpea flour, rice flour, and chia seed hydrogel in the formulation of reduced-fat gluten-free cookies was found favorable in terms of cookie nutritional properties, technological performance, and sensory acceptability. The obtained gluten-free cookies had a lower fat content and higher protein content compared with commercially available gluten-free cookies. Furthermore, the produced cookies had a favorable fatty acid composition. In terms of mineral content, all the chia seed hydrogel-containing cookies could be labeled as “a source of Fe and K”, while the ones with the cocoa powder addition could also bear the nutrition claim “high in Zn and Mg”. Fat replacement with chia seed hydrogel resulted in cookies with preserved hardness, weight, eccentricity, and specific volume, indicating that the chia seed hydrogel addition did not disturb the cookie texture and acted as a structure-forming agent instead of replacing the amount of vegetable fat. The results of the sensory analysis confirmed that it is possible to apply chia seed hydrogel to produce reduced-fat cookies with sensory properties comparable to their full-fat counterpart and commercial cookies, and that they are more appealing than commercial reduced-fat gluten-free cookies. Overall, the current results suggest that among the analyzed samples, sample CHC2 can be considered the best formulation as it provides reduced-fat gluten-free cookies with the highest content of proteins and minerals without compromising sensory acceptability. 

## 4. Materials and Methods

### 4.1. Materials

The following raw materials were used to prepare gluten-free cookies with reduced fat content: commercial gluten-free mixture (Nutry Allergy Center, Zemun, Serbia), chickpea flour (LeblebijaNaut—Besan, Bačko Gradište, Serbia), rice flour (Interpak, Kraljevo, Serbia), vegetable fat (palm-oil-based, melting range temperature 34–37 °C, Dijamant, Zrenjanin, Serbia), chia seeds (Perfect Day EuroCompany99 d.o.o., Ljubuški, Bosnia and Herzegovina), powdered sugar (Lučar, Novi Sad, Serbia), cocoa powder with reduced cocoa butter content (Dr. Oetker d.o.o., Šimanovci, Serbia), and vanilla and hazelnut aroma (Etol d.o.o., Celje, Slovenia). The commercial gluten-free mixture contained corn starch, corn flour, potato starch, rice flour, and guar gum. The chickpea flour had fat, saturated fatty acid, carbohydrate, sugar, fiber, and crude protein contents of 6.29, 0.78, 44.36, 4.41, 14.19, and 24.67%, respectively, while the rice flour had fat, saturated fatty acid, carbohydrate, sugar, and protein contents of 0.5, 0.1, 81.4, 0.1, and 7.8%, respectively. The fat, saturated fatty acid, carbohydrate, sugar, fiber, and protein contents of the chia seeds were 30.5, 3.2, 42.1, 0, 34.4, and 16.5%, respectively. The cocoa powder with reduced butter content (10–20% cocoa powder calculated on dry matter) had fat, saturated fatty acid, carbohydrates, sugar, and protein contents of 11.0, 7.0, 17.0, 3.0, and 24.0%, respectively.

Commercially available gluten-free cookies (Com 1—based on a mixture of buckwheat, maize, millet, and rice flours; Com 2—based on rice flour with the addition of plant proteins; Com 3—based on maize and rice flours with the addition of almond; and Com 4—based on millet flour with the addition of cocoa powder) served as conventional and functional benchmark samples.

### 4.2. Methods

#### 4.2.1. Chia Seed Hydrogel Preparation and Rheological Characterization

The chia seed gel rheological properties were examined in order to determine which concentration of gel (5, 8, and 10 g/100 g) will be used in the formulation of the gluten-free cookies. The preparation of gel included immersing a certain amount of intact chia seeds in 100 g of water at 37 °C and at a gelling time of 15 h. A HAAKE Mars rheometer (Thermo Scientific, Karlsruhe, Germany) was used to perform dynamic oscillatory tests. Mechanical spectra (frequency sweeps) were measured in the range 0.1–10 Hz at 1 Pa shear stress (previously determined, using “stress sweep” test, to be within the viscoelastic region). In order to prevent the slipping of the samples, a parallel plate system PP35 S (35 mm diameter, 1.5 mm gap) was used [36]. Analyses were performed at 20 °C in three replicates.

#### 4.2.2. Cookie Preparation

A raw material composition of cookie dough is shown in Table 5. A Control cookie, without chia seeds gel, was also prepared and further analyzed. The preparation of chia seed gel included sinking 8 g of chia seed into 100 g of water at a temperature around 37 °C with occasional stirring. The gelling time was 15 h.

After weighing, the powdered ingredients (gluten-free mixture, chickpea and rice flour, salt, baking powder, and cocoa powder) were mixed together. The first phase of dough preparation involved mixing vegetable fat without/with chia seed gel and aromas following the addition of powdered sugar and stirring for 1 min at a mixing speed of 5 (Gorenje, Slovenia). The next phase involved the gradual addition of powdered ingredients and mixing over a period of one minute. 

The obtained dough was processed on a dough sheeter (Mignon, Rimini, Italy) by gradually sheeting until the gap between the cylinders reached 4 mm. Subsequently, the dough was cut with the mold (round-shaped mold with a diameter of 45 mm). The shaped dough was baked for 12 min at 190 °C in a laboratory oven (MIWE Gusto CS, Arnstein, Germany). Afterwards, the cookies were cooled down at room temperature for 30 min and packed in polypropylene packages until further analysis.

#### 4.2.3. Chemical Composition of Gluten-Free Cookies

The proximate composition of the raw material and cookies was determined following AOAC [37] methods for moisture, protein, fat, crude fiber, and ash content. The available carbohydrates were obtained according to the difference obtained after subtracting the total fat, ash, crude fiber, and protein content from a 100 g basis mass. All the analyses were carried out in duplicate, and the results are expressed as g/100 g of sample weight.

#### 4.2.4. Fatty Acid Profile of Gluten-Free Cookies

A fatty acid profile of the gluten-free cookies was determined following a procedure published earlier [38]. A chloroform–methanol solution (2:1 ratio of chloroform to methanol) was used to extract the total lipids from samples, and the obtained extracts were dried by vacuum evaporation (40 °C). The solvent was evaporated under a steam of nitrogen and the residue was weighted. The 14% solution of boron (III)-fluoride in methanol was used to convert the extracted lipids into fatty acid methyl esters. The analysis of the samples was performed using GC Agilent 7890A (flame-ionization detector, auto injection module, fused silica capillary column DB WAX 30m, 0.25 mm, 0.50 µm). Helium was used as a carrier gas (purity over 99.9997 vol.%) with a flow rate of 1.26 mL/min. Fatty acids identification was performed by comparing the retention times of samples with those for standards (Supelco 37 Component Fatty Acid Methyl Ester Mix, Sigma-Aldrich, St. Louis, MO, USA). All analyses were performed in triplicates and the results were expressed as g/100 g of sample weight.

#### 4.2.5. Mineral Composition of Gluten-Free Cookies

The mineral content in gluten-free cookies was determined according to ISO 6869:2008 [39]. The samples were reduced to ashes (at 550 ± 15 °C) that were dissolved in HCl acid and atomized in the air via an acetylene flame. An atomic absorption spectrophotometer (SpectrAA-10, Varian Inc., Mulgrave, Victoria, Australia) was used to determine the mineral content in the samples. The analyses were performed in three replicates. 

#### 4.2.6. Textural Properties of Gluten-Free Cookies

The determination of the cookies’ texture was carried out using a Texture Analyzer (TA.XT2, Stable Micro Systems, Surrey, England). The analyzer was equipped with a 30 kg load cell and a 3-point bend rig (HDP/3PB). The cookie samples were placed on a specialist attachment (supports rig) with a 20 mm gap length. The compression mode was used with a travel distance of 8 mm and cross head speed of 3 mm/s. The maximum force was registered as a measure of cookie hardness.

#### 4.2.7. Color Measurement of Gluten-Free Cookies

The color of the gluten-free cookies’ upper surface was measured instrumentally by using a CR400 colorimeter (Konica Minolta Co., Osaka, Japan), and the results are shown according to the CIElab color system as lightness (L*), degree of redness or greenness (depending on a* > 0 or a* < 0), and degree of yellowness or blueness (depending b* > 0 or b* < 0).

#### 4.2.8. Physical Properties of Gluten-Free Cookies

Gluten-free cookies were evaluated for physical characteristics including the weight (g), diameters (D1 and D2, perpendicular to each other), and thicknesses of the baked cookies. The dimensional properties of the cookies were measured with a Vernier caliper using 10 cookies randomly chosen from the batch of cookies. The ratio between D1 and D2 was used as a measure of the cookies’ eccentricity, while the specific volume (cm^3^/g) was measured according to the AACC [40].

#### 4.2.9. Sensory Acceptability

The sensory acceptability of the gluten-free cookies was determined by untrained panelists (32 women and 18 men, ages 25 to 55) recruited from staff at the Institute of Food Technology (University of Novi Sad, Serbia). The overall acceptability and acceptability of the color, flavor, taste, and texture were scored using a 9-point scale labeled on the left with 1 = ‘‘dislike very much’’ and on the right with 9 = ‘‘like very much’’. Commercially available gluten-free cookies (Com 1–4) were included in the acceptability study as well. All the participants evaluated all the samples delivered in an odorless plastic container. The evaluation was performed in the laboratory for sensory analysis at the Institute of Food Technology equipped according to the ISO 8589:2007 (Sensory analysis—General guidance for the design of test rooms) [41]. 

The participants recruited for the acceptability study were informed about the study and those that agreed to participate in testing signed an informed consent form. The Ethics Committee of the Institute of Food Technology in Novi Sad, University of Novi Sad, Serbia, approved the study implementation (Ref. No. 175/I/28-3). 

#### 4.2.10. Statistical Analysis

All the analyses were carried out with the necessary number of measurements, and the mean values and standard deviations were calculated. The one-way ANOVA followed by Tukey’s multiple comparison test at a 95% confidence level (*p* < 0.05) was performed in order to determine the statistical differences among the samples. The statistical analysis was performed using the software package XLSTAT 2018.7. (Addinsoft, New York, NY, USA).

## Figures and Tables

**Table 1:** Chemical composition (g/100 g) and energy value (kcal) of developed reduced-fat gluten-free cookies (CH, CH1, CH2) compared with full-fat cookie (Control) and commercial (Com 1–4) samples.

Parameter	Com 1	Com 2	Com 3	Com 4	Control	CH	CHC1	CHC2
Moisture	n.a.	n.a.	n.a.	n.a.	4.56 ± 0.08 ^b^	5.95 ± 0.13 ^a^	3.81 ± 0.01 ^c^	4.30 ± 0.01 ^b^
Crude protein	4.5	13.23	3.67	5.25	9.32 ± 0.06 ^b^	9.67 ± 0.02 ^ab^	9.38 ± 0.01 ^b^	9.93 ± 0.05 ^a^
Fat	11.2	25.07	22.43	32.25	15.64 ± 0.06 ^a^	14.83 ± 0.02 ^b^	15.32 ± 0.01 ^a^	12.77 ± 0.03 ^c^
Saturated fatty acid	1.5	17.07	9.77	n.a.	7.25 ± 0.08 ^a^	6.34 ± 0.10 ^b^	6.57 ± 0.11 ^b^	5.37 ± 0. 08 ^c^
Carbohydrate	79.6	40.28	61.4	57.4	68.71 ± 0.13 ^a^	67.31 ± 0.17 ^b^	68.68 ± 0.02 ^a^	70.02 ± 0.14 ^a^
Sugar	16.3	24.32	2.8	n.a.	23.34 ± 0.11 ^b^	24.22 ± 0.29 ^a^	23.07 ± 0.03 ^b^	21.06 ± 0.17 ^c^
Dietary fiber	4.7	11.38	6.0	n.a.	0.66 ± 0.02 ^d^	0.91 ± 0.01 ^c^	1.33 ± 0.03 ^b^	1.45 ± 0.03 ^a^
Ash	n.a	n.a	n.a	n.a	1.12 ± 0.01 ^c^	1.32 ± 0.01 ^b^	1.48 ± 0.02 ^a^	1.52 ± 0.02 ^a^
Energy value	447	462	534	542	454	443	453	438

n.a.—not available; Com 1–4 refers to the commercially available cookie samples for which presented values are obtained from their nutrition labelling declarations. Data present mean value of replicates ± SD; different small letters within row indicate statistical significance (*p* < 0.05) according to the one-way ANOVA followed by Tukey’s HSD test.

**Table 2:** Fatty acid profile (g/100 g sample) of gluten-free cookies.

Fatty Acid	Control	CH	CHC1	CHC2
C12:0	0.05 ± 0.02 ^a^	0.04 ± 0.03 ^ab^	0.04 ± 0.01 ^ab^	0.03 ± 0.01 ^b^
C14:0	0.14 ± 0.04 ^a^	0.12 ± 0.05 ^b^	0.12 ± 0.01 ^b^	0.10 ± 0.01 ^c^
C16:0	6.12 ± 0.10 ^a^	5.52 ± 0.04 ^b^	5.58 ± 0.1 ^b^	4.55 ± 0.04 ^c^
C16:1	-	-	-	-
C18:0	0.94 ± 0.05 ^a^	0.66 ± 0.01 ^c^	0.82 ± 0.01 ^b^	0.69 ± 0.03 ^c^
C18:1t	-	-	-	-
C18:1c	5.68 ± 0.08 ^b^	5.80 ± 0.06 ^ab^	5.97 ± 0.08 ^a^	4.80 ± 0.04 ^c^
C18:2t	-	-	-	-
C18:2c (LA)	2.12 ± 0.06 ^b^	2.19 ± 0.02 ^b^	2.25 ± 0.05 ^a^	2.03 ± 0.03 ^c^
C20:0	-	-	-	-
C18:3n3 (LNA)	0.48 ± 0.03 ^b^	0.50 ± 0.03 ^b^	0.53± 0.04 ^ab^	0.57± 0.01 ^a^
C20:1	-	-	-	-
C22:0	-	-	-	-
C20:5n3	-	-	-	-
C23:0	-	-	-	-
C24:0	-	-	-	-
MUFA	5.68	5.80	5.97	4.80
PUFA	2.60	2.69	2.78	2.60
UFA (MUFA + PUFA)	8.28	8.49	8.75	7.40
SFA	7.25	6.34	6.57	5.37
PUFA/SFA	0.36	0.42	0.42	0.48

Data present mean value of three measurements ± SD; different small letters within row indicate statistical significance (*p* < 0.05) according to the one-way ANOVA followed by Tukey’s HSD test. MUFA—monounsaturated fatty acid; PUFA—polyunsaturated fatty acid; UFA—unsaturated fatty acid (total); SFA—saturated fatty acid.

**Table 3:** Mineral content (mg/100 g) and nutritional reference value (NRV) of gluten-free cookies.

Cookie Formulation	Mineral Content (mg/100 g)					
	**Na**	**K**	**Ca**	**Mg**	**Fe**	**Zn**
Control	362.2 ± 7.1 ^c^	362.2 ± 5.6 ^c^	44.6 ± 0.38 ^c^	45.8 ± 0.60 ^c^	2.01 ± 1.1 ^c^	12.8 ± 0.75 ^c^
CH	378.9 ± 5.3 ^b^	389.7 ± 8.2 ^b^	51.7 ± 0.38 ^b^	52.2 ± 0.80 ^c^	2.24 ± 0.90 ^c^	13.4 ± 0.30 ^c^
CHC1	357.1 ± 27 ^c^	396.5 ± 1.8 ^b^	55.1 ± 1.4 ^b^	64.7 ± 0.50 ^b^	3.28 ± 1.3 ^b^	15.4 ± 1.3 ^b^
CHC2	390.5 ± 12 ^a^	419.8 ± 11 ^a^	60.9 ± 1.2 ^a^	72.3 ± 2.7 ^a^	3.72 ± 0.40 ^a^	18.1 ± 0.80 ^a^
NRV * (mg/day)	-	2000	800	375	14	10

Data present mean value of three measurements ± SD; different small letters within row indicate statistical significance (*p* < 0.05) according to the one-way ANOVA followed by Tukey’s HSD test. * NRV—daily nutrient reference values for minerals for adults according to REGULATION (EU) No. 1169/2011 OF THE EUROPEAN PARLIAMENT AND OF THE COUNCIL of 25 October 2011 on the provision of food information to consumers [32].

**Table 4:** Color parameters and physical properties of gluten-free cookies.

Parameter	Control	CH	CHC1	CHC2
L*	74.02 ± 1.2 ^a^	76.78 ± 1.0 ^a^	49.87 ± 0.73 ^b^	50.59 ± 1.9 ^b^
a*	2.08 ± 0.48 ^b^	2.14 ± 0.33 ^b^	12.39 ± 0.23 ^a^	12.41 ± 0.50 ^a^
b*	29.92 ± 0.53 ^a^	30.98 ± 0.87 ^a^	23.56 ± 0.19 ^b^	24.18 ± 0.47 ^c^
Mass of cookie (g)	7.32 ± 0.24 ^a^	7.62 ± 0.15 ^a^	7.53 ± 0.23 ^a^	7.52 ± 0.26 ^a^
Specific volume (cm^3^/g)	5.52 ± 0.18 ^a^	5.41 ± 0.14 ^a^	5.27 ± 0.32 ^a^	5.18 ± 1.3 ^a^
Eccentricity	1.00 ± 0.01 ^a^	1.00 ± 0.01 ^a^	0.99 ± 0.01 ^a^	1.00 ± 0.01 ^a^

Data present mean value of replicated measurements ± SD; different small letters within row indicate statistical significance (*p* < 0.05) according to the one-way ANOVA followed by Tukey’s HSD test.

**Table 5:** Formulation of cookies.

Ingredients (g/100g)	Control	CH	CHC1	CHC2
Gluten-free flour mixture (g)	20	20	20	20
Chickpea flour (g)	40	40	40	40
Rice flour (g)	40	40	40	40
Sugar (g)	35	35	35	35
Vegetable fat (g)	25	20	20	15
Chia gel (g)	-	40	40	45
Cocoa powder (g)	-	-	5	5
Salt (g)	1	1	1	1
Vanilla aroma (g)	0.3	0.3	0.3	0.3
Hazelnut aroma (g)	0.2	0.2	0.2	0.2
Baking powder (g)	0.5	0.5	0.5	0.5
Water (g)	40	-	-	-

## Data Availability

Not applicable.

## References

[1] Wu J.H.Y., Neal B., Trevena H., Crino M., Stuart-Smith W., Faulkner-Hogg K., Yu Louie J.C., Dunford E. (2015). Are Gluten-Free Foods Healthier than Non-Gluten-Free Foods? An Evaluation of Supermarket Products in Australia. Br. J. Nutr..

[2] Melini V., Melini F. (2019). Gluten-Free Diet: Gaps and Needs for a Healthier Diet. Nutrients.

[3] Pang G.C., Xie J.B., Chen Q.S., Hu Z.H. (2012). How Functional Foods Play Critical Roles in Human Health. Food Sci. Hum. Wellness.

[4] Jnawali P., Kumar V., Tanwar B. (2016). Celiac Disease: Overview and Considerations for Development of Gluten-Free Foods. Food Sci. Hum. Wellness.

[5] Sandri L.T.B., Santos F.G., Fratelli C., Capriles V.D. (2017). Development of Gluten-Free Bread Formulations Containing Whole Chia Flour with Acceptable Sensory Properties. Food Sci. Nutr..

[6] Menga V., Amato M., Phillips T.D., Angelino D., Morreale F., Fares C. (2017). Gluten-Free Pasta Incorporating Chia (*Salvia Hispanica,* L.) as Thickening Agent: An Approach to Naturally Improve the Nutritional Profile and the in Vitro Carbohydrate Digestibility. Food Chem..

[7] Arslan M., Rakha A., Xiaobo Z., Mahmood M.A. (2019). Complimenting Gluten Free Bakery Products with Dietary Fiber: Opportunities and Constraints. Trends Food Sci. Technol..

[8] Martínez E., García-Martínez R., Álvarez-Ortí M., Rabadán A., Pardo-Giménez A., Pardo J.E. (2021). Elaboration of Gluten-Free Cookies with Defatted Seed Flours: Effects on Technological, Nutritional, and Consumer Aspects. Foods.

[9] Mancebo C.M., Picón J., Gómez M. (2015). Effect of Flour Properties on the Quality Characteristics of Gluten Free Sugar-Snap Cookies. LWT Food Sci. Technol..

[10] Sahagún M., Gómez M. (2018). Influence of Protein Source on Characteristics and Quality of Gluten-Free Cookies. J. Food Sci. Technol..

[11] Commission Implementing Regulation (EU) 2020/24 of 13 January 2020 Authorising an Extension of Use of Chia Seeds (Salvia Hispanica) as a Novel Food and the Change of the Conditions of Use and the Specific Labelling Requirements of Chia Seeds. http://data.europa.eu/eli/reg_impl/2020/24/oj.

[12] Sandoval-Oliveros M.R., Paredes-López O. (2013). Isolation and Characterization of Proteins from Chia Seeds (*Salvia Hispanica,* L.). J. Agric. Food Chem..

[13] Silveira Coelho M., de las Mercedes Salas-Mellado M. (2014). Chemical Characterization of Chia (*Salvia Hispanica,* L.) for Use in Food Products. J. Food Nutr. Res..

[14] Felisberto M.H.F., Wahanik A.L., Gomes-Ruffi C.R., Clerici M.T.P.S., Chang Y.K., Steel C.J. (2015). Use of Chia (*Salvia Hispanica,* L.) Mucilage Gel to Reduce Fat in Pound Cakes. LWT Food Sci. Technol..

[15] Hargreaves S.M., Zandonadi R.P. (2018). Flaxseed and Chia Seed Gel on Characteristics of Gluten-Free Cake. J. Culin. Sci. Technol..

[16] Pareyt B., Talhaoui F., Kerckhofs G., Brijs K., Goesaert H., Wevers M., Delcour J.A. (2009). The Role of Sugar and Fat in Sugar-Snap Cookies: Structural and Textural Properties. J. Food Eng..

[17] Shaltout O.E., Youssef M.M. (2007). Fat Replacers and Their Applications in Food Products: A Review. J. Food Sci. Technol..

[18] Pușcaș A., Tanislav A.E., Mureșan A.E., Fărcaș A.C., Mureșan V. (2022). Walnut Oil Oleogels as Milk Fat Replacing System for Commercially Available Chocolate Butter. Gels.

[19] Milićević N., Sakač M., Hadnađev M., Škrobot D., Šarić B., Hadnađev T.D., Jovanov P., Pezo L. (2020). Physico-Chemical Properties of Low-Fat Cookies Containing Wheat and Oat Bran Gels as Fat Replacers. J. Cereal Sci..

[20] Quilaqueo M., Iturra N., Contardo I., Millao S., Morales E., Rubilar M. (2022). Food-Grade Bigels with Potential to Replace Saturated and Trans Fats in Cookies. Gels.

[21] Kim Y.-J., Shin D.-M., Yune J.-H., Jung H.-S., Kwon H.-C., Lee K.-W., Oh J.-W., Kim B.-G., Han S.-G. (2022). Development of β-Cyclodextrin/Konjac-Based Emulsion Gel for a Pork Backfat Substitute in Emulsion-Type Sausage. Gels.

[22] Silav-Tuzlu G., Tacer-Caba Z. (2021). Influence of Chia Seed, Buckwheat and Chestnut Flour Addition on the Overall Quality and Shelf Life of the Gluten-Free Biscuits. Food Technol. Biotechnol..

[23] Punia S., Dhull S.B. (2019). Chia Seed (*Salvia Hispanica*, L.) Mucilage (a Heteropolysaccharide): Functional, Thermal, Rheological Behaviour and Its Utilization. Int. J. Biol. Macromol..

[24] Fernandes S.S., Salas-Mellado M., de las M. (2017). Addition of Chia Seed Mucilage for Reduction of Fat Content in Bread and Cakes. Food Chem..

[25] Capitani M.I., Corzo-Rios L.J., Chel-Guerrero L.A., Betancur-Ancona D.A., Nolasco S.M., Tomás M.C. (2015). Rheological Properties of Aqueous Dispersions of Chia (*Salvia Hispanica,* L.) Mucilage. J. Food Eng..

[26] Regulation (EC) No 1924/2006 of the European Parliament and of the Council of 20 December 2006 on Nutrition and Health Claims Made on Foods. http://data.europa.eu/eli/reg/2006/1924/2014-12-13.

[27] Costantini L., Lukšič L., Molinari R., Kreft I., Bonafaccia G., Manzi L., Merendino N. (2014). Development of Gluten-Free Bread Using Tartary Buckwheat and Chia Flour Rich in Flavonoids and Omega-3 Fatty Acids as Ingredients. Food Chem..

[28] Ciftci O.N., Przybylski R., Rudzińska M. (2012). Lipid Components of Flax, Perilla, and Chia Seeds. Eur. J. Lipid Sci. Technol..

[29] Kim S.L., Kim S.K., Park C.H. (2004). Introduction and Nutritional Evaluation of Buckwheat Sprouts as a New Vegetable. Food Res. Int..

[30] Culetu A., Ionescu V., Todasca M.C., Duta D.E. (2020). Evaluation of the Storage-Associated Changes in the Fatty Acid Profile of Oat-Based Gluten-Free Cookies Prepared with Different Fats. Food Sci. Biotechnol..

[31] Wood J.D., Enser M., Fisher A.V., Nute G.R., Sheard P.R., Richardson R.I., Hughes S.I., Whittington F.M. (2008). Fat Deposition, Fatty Acid Composition and Meat Quality: A Review. Meat Sci..

[32] Regulation (EU) No 1169/2011 OF the European Parliament and of the Council of 25 October 2011 on the Provision of Food Information to Consumers. http://data.europa.eu/eli/reg/2011/1169/2018-01-01.

[33] Zoulias E., Oreopoulou V., Tzia C. (2002). Textural Properties of Low-Fat Cookies Containing Carbohydrate- or Protein-Based Fat Replacers. J. Food Eng..

[34] Jan K.N., Panesar P.S., Singh S. (2018). Optimization of Antioxidant Activity, Textural and Sensory Characteristics of Gluten-Free Cookies Made from Whole Indian Quinoa Flour. LWT.

[35] Benkadri S., Salvador A., Zidoune M.N., Sanz T. (2018). Gluten-Free Biscuits Based on Composite Rice–Chickpea Flour and Xanthan Gum. Food Sci. Technol. Int..

[36] Ramos S., Fradinho P., Mata P., Raymundo A. (2017). Assessing Gelling Properties of Chia (*Salvia Hispanica,* L.) Flour through Rheological Characterization. J. Sci. Food Agric..

[37] The Association of Official Analytical Chemists (2000). AOAC Official Methods of Analysis.

[38] Pojić M., Mišan A., Sakač M., Dapčević-Hadnađev T., Šarić B., Milovanović I., Hadnađev M. (2014). Characterization of Byproducts Originating from Hemp Oil Processing. J. Agric. Food Chem..

[39] (2000). International Organization for Standardization Determination of the Contents of Calcium, Copper, Iron, Magnesium, Manganese, Potassium, Sodium and Zinc—Method Using Atomic Absorption Spectrometry.

[40] American Association of Cereal Chemist (2000). Approved Methods of the AACC, Method 10-05.

[41] (2007). International Organization for Standardization Sensory Analysis—General Guidance for the Design of Test Rooms.

